# FASTer diagnosis: Time to BEAT heart failure

**DOI:** 10.3399/BJGPO.2021.0006

**Published:** 2021-04-28

**Authors:** Clare J Taylor, Nick Hartshorne-Evans, Duwarakan Satchithananda, FD Richard Hobbs

**Affiliations:** 1 GP and NIHR Academic Clinical Lecturer, Nuffield Department of Primary Care Health Sciences, University of Oxford, Oxford, UK; 2 Clinical Board Member, Pumping Marvellous Foundation, Preston, UK; 3 Chief Executive and Founder, Pumping Marvellous Foundation, Preston, UK; 4 Consultant Cardiologist, University Hospitals of North Midlands, Preston, UK; 5 Professor and Head of Department, Nuffield Department of Primary Care Health Sciences, University of Oxford, Oxford, UK

**Keywords:** Heart failure, diagnosis, natriuretic peptide, testing, primary health care, general practice

## Introduction

Heart failure (HF) is common and costly.^1^ Around 26 million people are living with HF globally, and a substantial proportion of healthcare budgets is spent on HF services.^2^ Large trials have established the clinical and cost-effectiveness of drug and device therapies which have symptomatic and prognostic benefit.^3^ However, HF survival rates have not improved substantially in the last two decades.^4^ Timely diagnosis is important to allow initiation of treatments which can improve outcome. Lack of HF awareness, barriers in the diagnostic pathway, suboptimal use of HF medications, and a low number of HF nurse specialists are just some factors that may have contributed to lack of progress over time.^5^

In this article, we explain the importance of diagnosing HF, highlight current challenges for primary care, and propose a novel approach for raising awareness of HF amongst patients, carers, and GPs.

### Importance of diagnosis

Guidelines recommend that people with symptoms suggestive of HF presenting to primary care have a natriuretic peptide test, and referral for imaging and specialist review if the level is raised.^3^ However, a recent report by the British Heart Foundation highlighted that almost 80% of patients are diagnosed on emergency hospital admission, and called for earlier diagnosis in primary care.^5^

HF is a very treatable condition once diagnosed. It is categorised according to left ventricular ejection fraction: HF with reduced ejection fraction (HFrEF) and HF with preserved ejection fraction (HFpEF), and this distinction determines the most appropriate management pathway. There is a substantial evidence base for HFrEF treatments, which have been shown to improve quality of life, reduce hospitalisation, and increase survival.^3^ Prognostically beneficial treatments for HFpEF are more limited, but diagnosis remains important to explain symptoms and allow initiation of diuretic therapy to relieve fluid overload and intensification of treatment for existing risk factors.

### Challenges for primary care

The primary care part of the HF diagnostic pathway requires patients to recognise that there is a problem and seek medical help, then for GPs to consider the possibility of HF and refer appropriately.^6^ The main symptoms of HF are breathlessness, fatigue, and ankle swelling, which often develop gradually. A symptom such as breathlessness can have many possible causes including obesity, lack of exercise, or another medical condition, such as lung disease. The average age for HF diagnosis is 76 years and most patients have multimorbidity.^2^

We conducted a qualitative interview study of patients with a recent diagnosis of HF. Our data showed that it was common for older patients to attribute their symptoms to being a normal part of ageing, or to a pre-existing long term condition.^7^ Family members were often first to notice that something was wrong and were instrumental in facilitating the initial contact with healthcare services.^8^

When patients present to primary care, the diagnosis of HF then needs to be considered; common symptoms found in HF can be attributed to other conditions by clinicians too. Analysis of primary care data has shown that there is often a significant delay between initial presentation and referral to specialist services, with missed opportunities for earlier diagnosis and treatment.^9^ Currently, four in five people with HF are hospitalised around the time of diagnosis, which is distressing for patients, costly to the health service, and usually denotes progression to a later stage of disease.

If HF is suspected, guidelines recommend a natriuretic peptide test; then, if the level is raised, referral for echocardiography and cardiology assessment. Variable availability of natriuretic peptide testing, limited capacity in echocardiography services, and long waiting times for cardiology outpatient appointments are potential barriers in the pathway. Recent data also shows in a cohort of patients referred for diagnostic assessment by their GP and seen by a cardiologist, only around 55% have a HF diagnosis.^10^ This means that almost half of patients will have an alternative diagnosis to explain their symptoms, further complicating the process and highlighting symptom overlap with other conditions such as lung disease.

### Raising awareness through BEAT-HF

‘Heart failure’ is a frightening term and public awareness of HF as a condition is limited. HF has traditionally received less attention than other cardiovascular conditions, and this may partly contribute to delayed diagnosis. Images accompanying news reports about HF often feature a person clutching the middle of their chest which would be more representative of myocardial infarction.

It is important that patients and the public are aware of the symptoms of HF to be able to recognise the condition and seek help. Campaigns to prompt early action in serious illness have been a feature of previous public health interventions. Perhaps the most notable of these is the ‘Act FAST’ campaign to raise awareness of stroke symptoms. The FAST acronym stands for Face, Arms, Speech, then Time to call 999. The evidence for the effectiveness of the ‘Act FAST’ campaign is positive: patients, carers, and GPs reported awareness of the campaign, and an ability to distinguish stroke symptoms and take appropriate action.^11^

**Figure 1.**
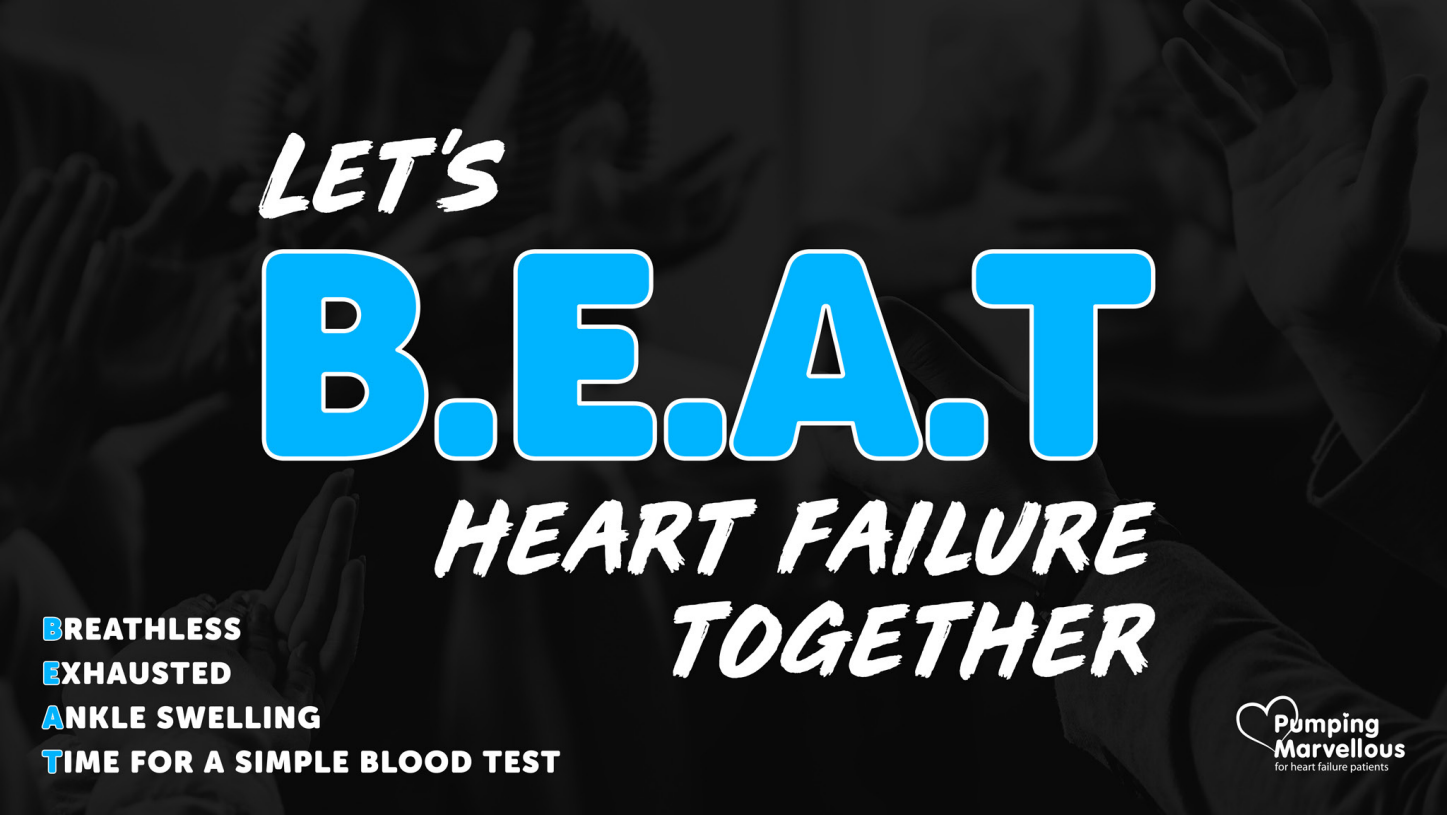
BEAT-HF acronym (Pumping Marvellous Foundation social media campaign)

Inspired by FAST, we propose the BEAT-HF acronym to help patients, the public, and GPs to consider the possibility of HF. BEAT stands for Breathless, Exhausted, Ankle swelling, Time for a simple blood test (Figure 1). These three key symptoms in combination are common in people with HF. We conducted a diagnostic accuracy study of patients presenting with symptoms suggestive of HF to primary care.^12^ Our results showed that, in the patients diagnosed with HF, 80% reported breathlessness, 70% had fatigue, and 84% had ankle swelling.

### Success of the BEAT-HF campaign

The patient-led HF charity the Pumping Marvellous Foundation launched #BEATHF on Twitter in 2020. The initial tweet proposing the acronym has over 500 likes and retweets. We now hope that BEAT-HF will be adopted more widely in the healthcare sector, particularly amongst GPs, so clinicians consider HF as a diagnosis for anyone presenting with breathlessness, exhaustion, and ankle swelling.

## Conclusion

Diagnosis is the key to treatment for people with HF. By raising awareness amongst patients, carers, and the wider public using BEAT-HF, people with symptoms may be more likely to see their doctor. Promoting BEAT-HF within primary care could prompt GPs to consider a diagnosis of HF, and test and refer at an earlier stage. By targeting these key points in the diagnostic pathway, this simple acronym may facilitate timely diagnosis, prevent costly hospital admissions, and allow earlier initiation of lifesaving treatments.
